# Sex Differences in Colorectal Cancer: Epidemiology, Risk Factors, and Clinical Outcomes

**DOI:** 10.3390/jcm14155539

**Published:** 2025-08-06

**Authors:** Sophia Tsokkou, Ioannis Konstantinidis, Menelaos Papakonstantinou, Paraskevi Chatzikomnitsa, Eftychia Liampou, Evdokia Toutziari, Dimitrios Giakoustidis, Petros Bangeas, Vasileios Papadopoulos, Alexandros Giakoustidis

**Affiliations:** 1 First Department of Surgery, General Hospital Papageorgiou, Aristotle University of Thessaloniki, 56429 Thessaloniki, Greece; menelaospap.md@gmail.com (M.P.); voula.hatzikomnitsa@yahoo.gr (P.C.); liampouef7@yahoo.gr (E.L.); evdo-t@hotmail.com (E.T.); dgiak@auth.gr (D.G.); pbangeas@gmail.com (P.B.); papadvas@auth.gr (V.P.); 2 Laboratory of Histology-Embryology, Department of Medicine, Faculty of Health Sciences, Aristotle University of Thessaloniki, 54124 Thessaloniki, Greece; ikonsc@auth.gr

**Keywords:** sex differences, colorectal cancer, epidemiology, risk factors, clinical outcomes, gender disparities, biological mechanisms, screening and prevention

## Abstract

Colorectal cancer (CRC) constitutes a major global health concern, ranking as the third most common cancer and the second leading cause of cancer-related mortality. The current review explores sex-based differences in CRC epidemiology, risk factors, tumor biology, and clinical outcomes. Males exhibit a higher incidence and mortality rate, with left-sided (distal) CRC predominating, while females are more frequently diagnosed with right-sided (proximal) tumors, which tend to be more aggressive and less responsive to conventional chemotherapy. Genetic disparities, including microsatellite instability and X-chromosome tumor suppressor genes, contribute to sex-specific differences in tumor progression and treatment response. Immune variations also influence disease outcomes, with females exhibiting stronger immune surveillance but higher exhaustion markers. Lifestyle factors such as body mass index (BMI), smoking, and hormonal influences further modulate CRC risk. While males are more vulnerable to obesity-related CRC, central obesity (waist-to-hip ratio) emerges as a stronger predictor in females. Additionally, smoking increases CRC risk differentially by tumor location. These findings underscore the importance of sex-specific approaches in CRC prevention, screening, and treatment, advocating for personalized medicine strategies tailored to gender-based biological and clinical distinctions.

## 1. Introduction

### 1.1. Epidemiology

Colorectal cancer (CRC) is one of the most significant global health concerns when it comes to malignancies. The World Health Organization (WHO) CRC is ranked as the third most commonly diagnosed cancer globally and the second leading cause of cancer-related mortality. Based on the International Agency for Research on Cancer (IARC), it is estimated that approximately 1.9 million new cases of CRC and more than 900,000 deaths are attributed to CRC annually [1]. Except for thyroid malignancies, non-reproductive tumors occur more frequently in males, with mortality rates twice as high as in females. Sex not only influences the incidence of malignancies but also clinicopathological disease features, treatment differences, therapeutic outcomes, and tolerability. The sex-associated differences are known as sexual dimorphisms [2]. Sexual dimorphism has been observed in CRC patients as it is debated to be influenced by both genders, suggesting that his difference may stem from variations in sex steroid hormone levels and gut microbiome composition [3]. Cancer statistics show that between 2015 and 2019, the average yearly CRC incidence rate was 33% higher in males, and their overall mortality rate was 43% higher than that of females [4]. Greater awareness of how gender can impact CRC risk can lead to new insights on how improvements in prevention, early diagnosis, treatment, and survival can be made [5].

### 1.2. Objective

The objective of this study is to examine sex-based differences in CRC across various aspects, including epidemiology, risk factors, tumor biology, and clinical outcomes. The research aims to determine how biological and lifestyle differences between men and women influence CRC incidence, progression, and response to treatment. By analyzing sex-specific disparities, the review seeks to summarize prevention strategies and support personalized treatment approaches for CRC patients.

## 2. Risk Factors for the Development of Colorectal Cancer

### 2.1. Modifiable and Non-Modifiable Risk Factors

A case-control study by Lewandowska A. et al., 2022, involving 800 participants, was conducted to assess risk factors, including both modifiable and non-modifiable contributors [6]. Among the non-modifiable factors, aging and hereditary predisposition were strongly associated with CRC development, with inflammatory bowel diseases such as ulcerative colitis and Crohn’s disease significantly increasing the risk for CRC [6].

Modifiable risk factors included obesity, low physical activity, smoking, and dietary habits such as high-fat and red meat consumption. Obese individuals with a BMI greater than 30 had a 1.27 times higher risk, and smokers faced a 2.17 times increased likelihood of CRC diagnosis. Additionally, reduced metabolic activity was found to be a significant contributor to cancer risk. The study emphasized the importance of maintaining a healthy lifestyle through balanced nutrition, regular exercise, and the avoidance of tobacco and excessive alcohol consumption. Socioeconomic disparities, including education and living conditions, also played a role in CRC incidence [6].

### 2.2. Diet-Related Behaviors and Nutritional Exposures in Colorectal Cancer Risk

Given CRC’s classification as a digestive system malignancy, its sensitivity to dietary exposures warrants closer attention. Evidence from studies illustrates the multifaceted role of diet in CRC risk modulation.

A great example of this matter is a scoping review of which 25 studies were included between the years of 2011 and 2021 that found dietary behaviors—particularly frequent consumption of red and processed meats, sugar-sweetened beverages, and high-fat diets, consistently correlated with elevated CRC risk. The risk was further enhanced by cooking methods that generate carcinogenic compounds, including grilling and pan-frying, whereas rare-cooked meats appeared protective. Meal frequency emerged as another factor, with higher frequencies linked to reduced risk, especially in metabolically active individuals [7].

Building upon this, a large-scale prospective study involving 542,778 women in the UK further confirmed these associations while evaluating nearly 100 dietary variables. Alcohol and red/processed meat intake were reaffirmed as risk enhancers, whereas calcium (particularly from dairy sources) emerged as a potent protective nutrient, showing a 17% reduced risk per 300 mg/day. Mendelian randomization findings supported a causal role for milk-derived calcium in CRC prevention. Additional inverse associations were observed for fruit, wholegrains, fiber, breakfast cereals, vitamin C, and folate, though some were attenuated after adjusting for confounding lifestyle and dietary factors [8].

### 2.3. Pharmacological Regimens and Risk for Colorectal Cancer Development (Table 1)

#### 2.3.1. Acid Suppressive Medications

Of particular relevance are the medications (non-chemotherapeutic regimens) that are associated with the risk of CRC development. The study of Chubak J. et al., 2009 [9] discusses acid suppressive medications, such as proton pump inhibitors (PPIs) and histamine receptor antagonists (H2 blockers), that are commonly used to treat gastrointestinal (GI) disorders. Laboratory studies have suggested that the prolonged use of these medications can elevate gastrin levels (a hormone linked to increased cell proliferation in the colon) and potentially raise the risk for CRC development [9]. In a population-based case-control study conducted in Washington State, researchers evaluated 641 patients with CRC alongside 641 matched controls to investigate this association. The results indicated that exclusive PPI use was modestly associated with CRC risk (OR = 1.7; 95% CI = 0.8–4.0), though the small number of exposed individuals made the result inconclusive, allowing room for debate and further research on the matter for clarification. In contrast, H2 blocker use alone showed no significant relationship with CRC risk (OR = 0.8; 95% CI = 0.6–1.1), and combined use of PPIs and H2 blockers did not demonstrate an increased risk (OR = 0.9; 95% CI = 0.5–1.4) [9]. Moving on, a recent prospective analysis conducted by Babic A. et al., 2020 [10], did not support a causal relationship between acid-suppressive medication use and CRC development, providing reassuring evidence for long-term PPI and H2RA users. More precisely, no significant association was found between baseline PPI use and CRC risk (HR = 0.89; 95% CI, 0.71–1.12), and no increased risk was observed for long-term PPI use (lagged 8–10 years: HR = 1.12; 95% CI, 0.78–1.59). The H2RA use at baseline was associated with lower CRC risk (HR = 0.76; 95% CI, 0.60–0.95), but this trend was not consistent over longer durations of use. Finally, no significant association was observed for the duration of PPI or H2RA use, indicating that prolonged medication exposure did not lead to higher CRC risk [10].

**Table 1:** Pharmacological Regimens and Association with Colorectal Cancer Risk (CRC)—This table provides a comparative overview of different medication types, highlighting their potential impact on CRC risk and relevant clinical insights.

Medication Type	Examples	Impact on CRC Risk	Notable Findings
Acid Suppressants	PPIs (Omeprazole, Lansoprazole)	Minimal risk; weak association with CRC	No causal link confirmed; debated over long-term use
Anti-Diabetics	Metformin, Sulfonylureas (Glimepiride, Gliclazide)	Sulfonylureas increase CRC risk, especially in older patients	Metformin shows no significant impact on CRC risk.
Antibiotics	Penicillins, Quinolones, Cephalosporins, Metronidazole	Broad-spectrum antibiotics associated with increased CRC risk	Long exposure (>30 days) linked to heightened risk

#### 2.3.2. Anti-Diabetic Medication

Furthermore, a large number of older people suffering from DM are under medication. Thus, antidiabetic medications are essential and necessary to be under investigation for the development and contribution to adverse outcomes. A population-based nested case-control study conducted by Shin C.M. et al. in 2020 [11] explored the association between anti-diabetic medications and CRC risk in patients with type 2 DM (T2DM) using South Korea’s National Health Insurance Corporation database. The study analyzed 4228 CRC cases and 4228 matched controls. A crucial finding of the study was that sulfonylurea use significantly increased the risk of CRC, with an adjusted odds ratio (aOR) of 1.14 (95% CI: 1.05–1.25). The risk appeared to rise in correlation with cumulative doses, suggesting a dose-dependent relationship (*p* = 0.0008). In contrast, metformin, thiazolidinediones, meglitinides, dipeptidyl peptidase-4 inhibitors (DPP-4), and α-glucosidase inhibitors showed no significant impact on CRC risk. A more detailed analysis of individual sulfonylureas revealed that gliclazide was associated with a lower CRC risk (aOR = 0.85; 95% CI: 0.72–1.00; *p* < 0.05), while glimepiride significantly increased the risk (aOR = 1.14; 95% CI: 1.06–1.22). The results indicate that not all sulfonylureas are equally risky for CRC, with gliclazide potentially providing a protective effect in comparison to other drugs in the same class. Furthermore, age appeared to be a significant factor in the risk of CRC among individuals who used sulfonylureas. The risk of sulfonylureas was substantially increased in older patients (≥65 years), while it was not statistically significant in younger patients (<65 years). This discovery underscores the significance of conducting patient-specific risk assessments when prescribing sulfonylureas to diabetic patients. It is intriguing that the risk of colorectal cancer was slightly elevated by the short-term use of DPP-4 inhibitors, which lasted between 180 and 360 days (aOR = 1.23; 95% CI: 1.04–1.45). Nevertheless, this trend was not maintained over the course of prolonged use, indicating that any potential CRC-promoting effects of DPP-4 inhibitors may be transient or influenced by confounding variables [11].

#### 2.3.3. Use of Antibiotics as a Possible Adverse Contributor

A systematic review and meta-analysis by Simin J. et al., 2020 [12] investigated the correlation between oral antibiotic use and CRC risk by examining 10 studies, encompassing 4.1 million individuals and 73,550 CRC cases. The study revealed a 17% increased CRC risk among antibiotic users (effect size (ES) = 1.17, 95% CI = 1.05–1.30), with broad-spectrum antibiotics showing the strongest correlation (ES = 1.70, 95% CI = 1.26–2.30). On the contrary, narrow-spectrum antibiotics exhibited no significant effect (ES = 1.11, 95% CI = 0.93–1.32). Certain antibiotic classes were linked to higher CRC risk, such as penicillins (ES = 1.16), quinolones (ES = 1.23), sulfonamides (ES = 1.17), cephalosporins (ES = 1.33), and metronidazole (ES = 1.28). While no clear dose-response relationship was established, CRC risk appeared to plateau after 30 days of cumulative antibiotic exposure, indicating that prolonged usage may not continuously increase cancer risk. Notably, risk patterns were similar for colon and rectal cancer, reinforcing the idea that antibiotic-related effects may not be tumor location-specific [12].

#### 2.3.4. Evaluating Statin Effectiveness

A population-based case-control study, combined with a meta-analysis of 48 studies, provided compelling evidence for a moderate chemoprotective effect of statins against CRC. Current statin use was associated with a 13% reduced risk of CRC, with protective effects emerging after six months and peaking between one and three years of continuous use. Significantly, the benefit did not persist after discontinuation or beyond three years, indicating a time-dependent and reversible effect. The analysis revealed stronger risk reduction with moderate and high-intensity statin regimens, and individual drugs like simvastatin and rosuvastatin showed significant associations. Protective effects were observed with both lipophilic and hydrophilic statins, and were more pronounced in individuals aged 70 or younger. Notably, the study identified a synergistic benefit when statins were combined with fibrates, suggesting an additive effect through complementary lipid-modulating and anti-inflammatory mechanisms. The meta-analysis further confirmed a consistent 10% reduction in CRC risk across diverse study designs, reinforcing the potential of statins as a chemopreventive strategy. These findings emphasize the relevance of statin type, intensity, duration, and co-medication context in optimizing CRC risk mitigation [13]. The study highlights that meaningful sex-based differences in the chemopreventive effects of statins on CRC are noted. While statins conferred a significant reduction in CRC risk for both sexes, the protective effect was notably stronger in males. Specifically, males experienced an overall 29% risk reduction, with protection against colon cancer reaching up to 59%, compared to 16% and 36% in females, respectively. These findings may reflect differences in drug metabolism, hormonal profiles, or lifestyle-related adherence patterns. Additionally, moderate-to-high intensity statin regimens and agents like simvastatin and rosuvastatin were particularly effective in younger male populations. Such distinctions outline the potential value of incorporating sex as a factor when evaluating statin use for CRC prevention and support the pursuit of tailored strategies in chemopreventive research [13].

Another recent meta-analysis published adds further weight to the evidence supporting statins’ chemopreventive role in CRC by synthesizing data from 30 high-quality cohort and case-control studies encompassing over 2.4 million individuals, the study found that statin use was associated with an 11% reduction in overall CRC risk (OR: 0.89; 95% CI: 0.84–0.95) and a 16% reduction in rectal cancer risk. Notably, the protective effect was more pronounced in younger age groups—22% risk reduction in individuals aged 50–59—and in men, who experienced a 29% reduction compared to 16% in women. The analysis also highlighted a stronger protective effect against colon cancer, with statin use reducing risk by 59% in men and 36% in women. These findings reinforce the hypothesis that statins may exert anticancer effects through cholesterol-lowering and anti-inflammatory mechanisms, and they underscore the importance of considering age and sex when evaluating statin-based prevention strategies. When integrated with previous studies, this paper strengthens the case for statins as a potential adjunct in CRC risk reduction, particularly in high-risk populations [14].

### 2.4. Ethnicity and Disparities for Colorectal Cancer Risk

Ethnicity influences CRC risk, with disparities in incidence, mortality, and access to screening across different populations. Studies show that Black, Hispanic, and Native American individuals often experience higher CRC mortality rates, partly due to delayed diagnosis and limited healthcare access. More specifically, the study of Birch R. J. et al., 2025 [15], which is among the most recently published studies, states that there is a significant disparity in early-onset CRC, tumor location, stage at diagnosis, and access to diagnostic pathways for different ethnic groups. Findings revealed that early-onset CRC (meaning diagnosis was before the age of 50) was substantially more common among non-white populations, with Asian (17.9%), Black (15.5%), and Mixed/Multiple ethnic groups (21.8%) exhibiting higher rates compared to White individuals (5.5%). Differences in route to diagnosis were also observed as non-white individuals were less likely to be diagnosed via the expedited “2-week wait” referral system, with potentially delayed treatment initiation. Tumor location varied per ethnicity, with rectal cancer being most prevalent among Asian individuals (39.2%), while Black individuals had the highest proportion of right-sided colon cancer (39.9%). Regarding disease progression, Black patients faced higher odds of late-stage (Stage IV) CRC at diagnosis (odds ratio (OR) = 1.18), whereas Asian patients were less likely to be diagnosed at Stage IV (OR = 0.82) compared to their White counterparts. Socioeconomic disparities were evident, as over 55% of Asian and 69% of Black CRC patients resided in deprived areas, potentially impacting healthcare access and screening rates. These findings highlight the urgent need for ethnic-specific interventions to enhance CRC screening participation, improve timely referrals, and address disparities in healthcare accessibility to reduce late-stage diagnoses among minority populations [15].

### 2.5. Genetic Predisposition and Syndromes Associated with Colorectal Cancer

Several factors suggest a genetic contribution to CRC. These include (1) a significant family history of CRC and/or polyps; (2) the occurrence of multiple primary cancers in a patient with CRC; (3) the presence of other cancers within the family that align with known hereditary syndromes conferring an increased risk of CRC, such as endometrial cancer; and (4) an early age of CRC diagnosis. Although two syndromes (MUTYH-associated polyposis and NTHL1) are inherited in an autosomal recessive pattern, hereditary CRC is most frequently inherited in an autosomal dominant pattern. The probability that an individual with cancer possesses a pathogenic variant in a mismatch repair (MMR) gene linked to Lynch syndrome, the most prevalent inherited CRC syndrome, can be estimated using at least three validated computer models. These prediction models consist of the MMRpro, MMRpredict, and PREMM5 (Prediction Model for gene Mutations). Genetic evaluation, referral, and testing are recommended for individuals with a quantified risk of 2.5% or greater on PREMM5 or 5% or greater on MMRpro and MMRpredict [16].

Hereditary CRC is characterized by two well-described forms. The polyposis, which includes familial adenomatous polyposis (FAP) and attenuated FAP (AFAP), is caused by pathogenic variants in the APC gene. The MUTYH-associated polyposis, caused by pathogenic variants in the MUTYH gene, and secondly, the Lynch syndrome—often referred to as hereditary nonpolyposis CRC, which is caused by germline pathogenic variants in the DNA MMR genes (MLH1, MSH2, MSH6, and PMS2) and EPCAM. Other CRC syndromes and their associated genes include oligopolyposis (POLE, POLD1), NTHL1, juvenile polyposis syndrome (BMPR1A, SMAD4), Cowden syndrome (PTEN), and Peutz-Jeghers syndrome (STK-11). Extracolonic malignancies and other manifestations are also associated with numerous of these syndromes. The genetic basis of serrated polyposis syndrome, which is distinguished by the presence of hyperplastic polyps, is unknown; however, it is suspected to have a familial component. The natural history of certain syndromes is still being described. Additional families demonstrate the aggregation of CRC and/or adenomas, but lack an apparent correlation with a specific hereditary syndrome. These families are collectively referred to as familial CRC. Furthermore, the majority of individuals with CRC diagnosed prior to the age of 50 and who do not have a family history of cancer do not possess a pathogenic variant that is linked to an inherited cancer syndrome [16].

## 3. Sex-Based Differences in Colorectal Cancer

### 3.1. Sex-Based Tumor Suppressor Gene Expression and Cancer Risk

A sex-based disparity in cancer incidence has been observed, with males experiencing a 20.4% higher overall cancer risk compared to females, even after adjusting for environmental factors such as tobacco exposure and socioeconomic influences. Researchers have identified a mechanism in which certain tumor-suppressor genes (TSGs) on the X chromosome escape X-inactivation in females, allowing them to retain functional copies even after mutation. On the other hand, males only possess one X chromosome, lacking the protective redundancy, making them susceptible to mutations in these genes. Six “Escape from X-Inactivation Tumor Suppressor” (EXITS) genes were identified, including *ATRX*, *CNKSR2*, *DDX3X*, *KDM5C*, *KDM6A*, and *MAGEC3*. They significantly displayed higher mutation frequencies in male cancers across multiple tumor types. Additionally, Y-chromosome loss in males has been observed in certain cancers, further diminishing the protective effects of Y-linked gene homologs, potentially increasing cancer susceptibility. These findings suggest that male and female cancers may exhibit distinct genetic profiles, influencing tumor biology, progression, and drug response. Recognizing these sex-specific genetic vulnerabilities is essential for developing targeted cancer prevention, screening, and treatment strategies, emphasizing the need for personalized medicine approaches that consider gender-based tumor genetics and molecular pathways [2,17].

### 3.2. Sex-Based Immune Differences in Colorectal Cancer Tumor Microenvironment

**Figure 1:**
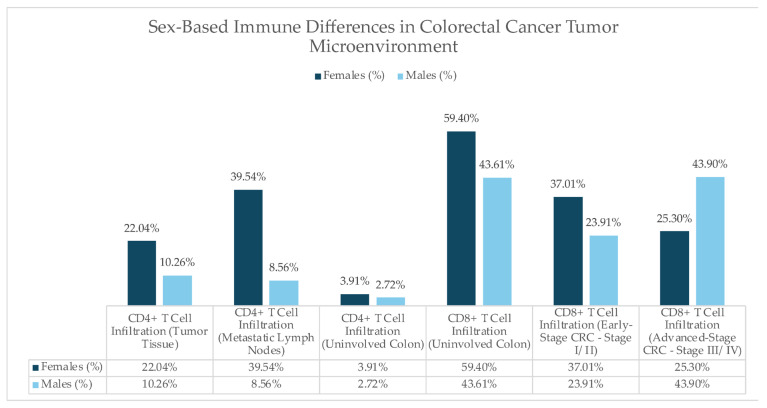
Sex-Based Immune Differences in Colorectal Cancer Tumor Microenvironment. This figure illustrates the comparative analysis of CD4+ and CD8+ T cell infiltration in tumor tissue, metastatic lymph nodes, and uninvolved colon between female and male colorectal cancer (CRC) patients.

Moreover, CRC is greatly influenced by the tumor microenvironment (TME). The TME consists of immune cells, fibroblasts, vascular structures, and extracellular components, all of which interact with tumor cells, shaping the disease outcomes. Particularly, the study of Geddes A.E. et al., 2022 [18], explored the sex-based differences in the TME in CRC. The study revealed significant disparities in immune cell infiltration, gene expression, and survival outcomes. Females with CRC exhibited higher CD4+ T cell infiltration in tumor tissue (22.04% vs. 10.26%), metastatic lymph nodes (39.54% vs. 8.56%), and uninvolved colon (3.91% vs. 2.72%). CD8+ T cells were also more prevalent in the uninvolved colon of females (59.40% vs. 43.61%), and tumors in early-stage CRC (Stage I/II) had greater CD8+ infiltration compared to advanced-stage CRC (Stage III/IV) (37.01% vs. 23.91%). Higher CD8+ infiltration was correlated with improved survival (43.9 months vs. 25.3 months), although no significant survival differences were observed between males and females. Gene expression analysis showed sex-specific immune regulation. Females had higher expression of T cell activation genes (IL18R1, STAT4, GBP1, GZMK) but lower expression of regulatory T cell (Treg) markers (DAB2, TNFRSF25, LRRC32), suggesting reduced immune suppression. Exhaustion markers (PD-L1, TIGIT, FBP1) were elevated in tumors in females, indicating potential immune differences influencing the disease progression [18]. These sex-based differences in CRC TME are graphically represented in Figure 1 and summarized in Table 2.

### 3.3. Tumor Location and Gender Differences in Colorectal Cancer Patients

The clinical manifestation of CRC varies depending on the tumor location, stage, and individual patient factors. In younger patients, symptoms often go unrecognized or misattributed to benign conditions, leading to delayed diagnosis and poorer prognosis. The most common symptoms include abdominal pain (63%), changes in bowel habits (54%), rectal bleeding (53%), and unexplained weight loss (32%). Diarrhea is more frequently reported than constipation, and many patients experience multiple symptoms simultaneously [19]. A great proportion of patients (especially younger individuals) endure symptoms for 3 months or longer before seeking medical attention, contributing to advanced-stage diagnoses (Stage III–IV in 75% of cases) [19].

CRC exhibits distinct tumor location patterns between males and females and thus significantly influences the diagnosis and treatment. Generally, females are more likely to develop right-sided (proximal) CRC (RCRC), which occurs in the ascending colon and cecum and is often correlated to mutations in the DNA mismatch repair pathway, microsatellite instability (MSI), *BRAF* mutations, and the serrated pathway of carcinogenesis [20,21]. These tumors tend to be more aggressive and less responsive to traditional chemotherapy as they are diagnosed in advanced stages and larger tumors, which are often poorly differentiated [22]. Hormonal influences, particularly estrogen, may contribute to these differences, as younger females seem to have a protective effect against MSI before menopause, but their risk increases post-menopause [23]. Moreover, RCRC is generally harder to detect using stool-based screening tests like the fecal immunochemical test (FIT). The difficulty lies in the lower sensitivity for proximal tumors, leading to delayed diagnoses in many female patients and, thus, poor prognosis [24]. RCRC is usually associated with abdominal pain, anemia, and upper gastrointestinal symptoms [19]. On the right side of the colon near the cecum, cancers usually grow into the space within the colon. Right-sided colon cancers tend to be asymptomatic or cause symptoms only when they are at an advanced stage and fairly large, but they can become large enough to be painful. In these cases, anemia from chronic blood loss is often the first sign and is why a stool test for occult, or hidden, blood is important (guaiac) [25]. Some symptoms correlate with worse survival outcomes, including loss of appetite, back pain, and urinary symptoms, whereas rectal bleeding and longer symptom duration are linked to better prognosis [26].

On the contrary, males commonly develop left-sided (distal) CRC (LCRC), affecting the descending colon, sigmoid colon, and rectum. LCRC follows the CIN pathway, the traditionally proposed model, where carcinogenesis begins with the inactivation or deletion of adenomatous polyposis coli (APC) tumor suppressor gene, followed by the activation of Kirsten Ras homolog (KRAS) oncogene and the inactivation of p53 tumor suppressor gene. The Cancer Genome Atlas (TCGA) study confirms the model and proposes various mutations, including *APC*, *TP53*, *KRAS*, *PIK3CA*, *FBXW7*, *SMAD4*, *TCF7L2*, and *NRAS* as being the most frequently mutated genes in CIN tumors [20,22].

Tumor location influences symptom presentation. Generally, most polyps and cancers appear on the left side of the colon. In the left or descending colon, the channel is narrow, and the cancer usually grows around the colon wall and encircles it. Left-sided colon cancer typically constricts the bowel channel, resulting in partial blockage. For this reason, the typical symptomatology includes constipation, change in bowel habits, and narrow, ribbon-shaped stool when a cancer is low in the rectum [19,25]. On the LCRC, symptomatology also includes rectal bleeding.

Therapy responses are different among tumor entities. LCRC patients benefit more from adjuvant chemotherapies, including 5-fluorouracil (5-FU)-based regimes, and targeted therapies such as anti-epidermal growth factor receptor (EGFR) therapy, and have a better prognosis. RCRC patients do not respond well to conventional chemotherapies, but demonstrate more promising results with immunotherapies because these tumors have a high antigenic load [22]. FIT screening is more effective in identifying LCRC due to their tendency to bleed, making screening more beneficial for males [24].

**Figure 2:**
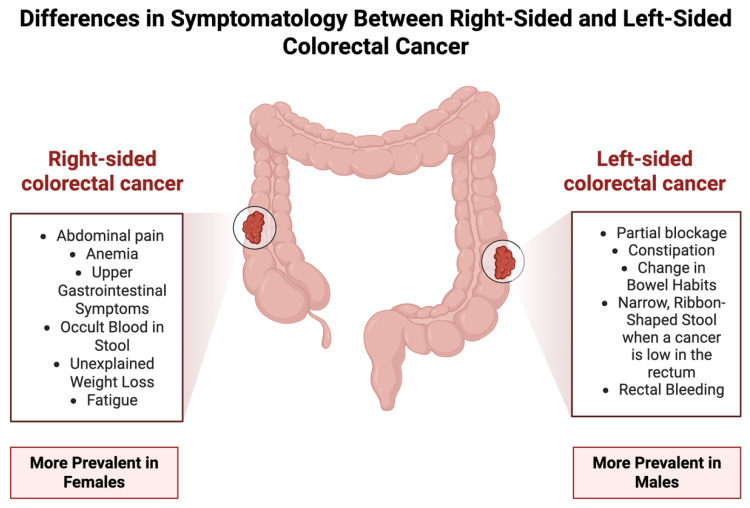
Right versus Left-Sided Colorectal Cancer—Understanding the Differences in Symptomatology and Presentation. This visual representation highlights key distinctions between right-sided and left-sided colorectal cancer, including symptoms and clinical characteristics [27].

These sex-based differences in tumor location and clinical manifestation of CRC are summarized in Figure 2 and Table 3 and Table 4.

### 3.4. Prevalence and Risk Factors Among Female and Male Populations

A population-based study published in 2023 by Erning F. N. et al. [28] revealed that over a 10-year duration (from 2010 to 2020), 147,394 patients were diagnosed with CRC in the Netherlands, with 101,405 cases of colon cancer (69%) and 45,989 cases of rectal cancer (31%). Across both groups, males were generally younger at the time of diagnosis compared to females, with a median age of 71 years and 72 years, respectively, in colon cancer, and 68 years and 69 years in rectal cancer [28]. Recent studies also highlight a high prevalence of CRC among younger individuals with a diagnosis of advanced stage [28,29]. Specifically, the incidence rate of CRC rises between 1% and 2% annually in individuals under the age of 55. This is an alarming trend since the mid-1990s. The mortality rate in young people has also increased by about 1% yearly since the mid-2000s. Alarmingly, CRC has emerged as the leading cause of cancer-related death in males under the age of 50 and as the second most common in females of the same age group. These trends highlight the urgency of enhanced screening and prevention strategies [30].

Based on Abotchie P.N. et al., 2012 [31], a comprehensive study examining sex differences in CRC incidence in the United States from 1975 to 2006 revealed that the incidence rate (IR) ratio between males and females was 1.38, indicating a substantially higher CRC incidence in males. This discrepancy persisted across all age stratifications, tumor sites, tumor stages, and geographical regions. Additionally, the study observed that CRC incidence increased dramatically with age, with the highest rates reported among individuals over the age of 70 [31]. However, even though there was a greater prevalence of CRC in the male population, female patients (over 65 years) tend to be diagnosed in a more advanced stage and have a more aggressive cancer type than males, thus decreasing the survival rate [32].

The observation for the advanced-stage diagnoses is elaborated by Choi Y. et al., 2024 [28], stating that right-sided (proximal) tumors occur predominantly in female and older patients and are associated with vague symptoms in comparison to left-sided colon cancers, that is more frequently diagnosed in males. Thus, the lack of symptomatology results in late diagnosis and delayed treatment initiation, resulting in poor outcomes. To elaborate further on the tumor location, prevalence, and notable sex differences, females with colon cancer had a higher proportion of right-sided tumors (59.2%), while males more frequently exhibited left-sided tumors (49.3%). Females additionally had a greater prevalence of poorly differentiated tumors (13.9%) compared to males (9.4%). The significantly higher MSI tumor rate in females (22.9%) compared to males (12.0%) suggests critical molecular differences in CRC biology. This could have profound implications for treatment approaches and responsiveness to immunotherapy [28].

#### 3.4.1. Body Mass Index (BMI)

Additionally, females were more likely to have a normal BMI, while males had a higher rate of overweight classification. The World Cancer Research Fund stated that an increase in BMI of around 5 kg/m^2^ raised the risk of CRC by 23% for males, but only 9% for females. On the contrary, an equivalent increase in waist-to-hip ratio raised the risk by 25% for females and only 5% for males [33]. Additionally, the study of Safizadeh F et al., 2024 [34], found that central obesity, measured by waist-to-hip ratio (WHR), is a significantly stronger predictor of CRC risk than general obesity, measured by body mass index (BMI). While BMI showed a weak association with CRC risk, its significance diminished after adjusting for WHR. In contrast, WHR maintained a strong correlation with CRC risk across all BMI categories, reinforcing its independent role as a key risk factor. Additionally, BMI was not associated with CRC risk in females or with rectal cancer after mutual adjustment, whereas WHR showed consistent associations in both sexes and across both colon and rectal cancer cases [34]. Furthermore, a nationwide population-based cohort study, Saeed U. et al., 2023 [35] found that elevated BMI was associated with a higher risk of CRC in the male population, where the strongest association was observed with right-sided colon cancer. In females, elevated BMI in early adulthood, meaning ages 16–29, was associated with an increased risk of both right- and left-sided colon cancer. Regarding cancer stage at diagnosis, elevated BMI in males was consistently associated with all stages of colon cancer, suggesting that increased body weight contributes to CRC risk regardless of disease severity. On the other hand, females with higher BMI were more likely to present with advanced-stage right-sided colon cancer. Interestingly, BMI was not significantly linked to CRC-specific mortality in males, but in females, higher BMI correlated with an increased risk of colon cancer death, particularly for left-sided colon cancer [35].

#### 3.4.2. Tobacco Smoking and Risk for Colorectal Cancer Development

Tobacco smoking is another contributing risk factor for CRC. This study of Gram I.T. et al., 2020 [36] examined the relationship between smoking and CRC risk, revealing distinct sex-specific differences in tumor location. Among male smokers, the risk of left (distal) colon cancer was 39% higher (HR = 1.39; 95% CI = 1.16–1.67) in comparison to never smokers, whereas right (proximal) colon cancer risk remained unchanged (HR = 1.03; 95% CI = 0.89–1.18). Moreover, female smokers showed a 20% increased risk of right colon cancer (HR = 1.20; 95% CI = 1.06–1.36), but no significant association when it comes to left colon cancer (HR = 0.96; 95% CI = 0.80–1.15). Additionally, females had a stronger link between smoking and rectal cancer, with an increased risk of 58% (HR = 1.58; 95% CI = 1.28–1.95) compared to 40% in men (HR = 1.40; 95% CI = 1.16–1.69). The study revealed that longer smoking duration and higher pack-years correlated with a greater CRC risk, with right colon cancer risk rising for females and left colon cancer risk increasing for males. Among postmenopausal females, both menopausal hormone therapy (MHT) users and non-users exhibited elevated risks of right colon cancer linked to smoking, suggesting possible hormonal interactions. These findings highlight that smoking is a confirmed risk factor for CRC, but differences in tumor location between males and females significantly impact both diagnosis and treatment strategies. Understanding these distinctions is crucial for personalized medicine approaches [36].

The reported sex-based differences in CRC prevalence and risk factors are summarized in Table 5.

#### 3.4.3. Pre-Existing Comorbidities

Comorbidity data revealed that females were less likely to have any pre-existing conditions, with 54.0% of females with colon cancer reporting no comorbidities compared to 46.2% of males [28]. Generally, older CRC patients commonly present with pre-existing comorbidities, including cardiovascular diseases (CVD), previously diagnosed malignancies—in either the large bowel or a distant organ. and hypertensive disorder (HD) (with a higher tendency among females) being the most common comorbidities. Chronic obstructive pulmonary disease (COPD) and diabetes mellitus (DM) were found to be prevalent to a lesser extent, according to Marco M. F. et al., 2000 [37]. A retrospective study conducted in Korea stated that 49.6% of colon cancer patients had equal to and more than three comorbidities [38]. Another observational cohort study conducted in the United States states that approximately 40% of CRC patients had 1 to 3 comorbidities and 19% had ≥4 comorbidities [39]. However, it is important to state that comorbidities differ in their associations with age at diagnosis and stage. A recent study revealed that dementia and chronic heart failure (CHF) were associated with older age patients, whereas inflammatory bowel disease (IBD) and alcohol use were associated with a younger onset of the disease [29]. Furthermore, the study of Gheybi 2020 [40] states that of 11,656 CRCs diagnosed during the years 2003 to 2013, a greater prevalence of comorbidities was observed in colon cancers rather than rectal cancer patients. The most prevalent comorbidities for colon and rectal cancers, respectively, included HD (25.9%, 22.0%), DM (17.3%, 15.6%), and gastric disease (11.4%, 12.4%) [40].

## 4. Screening of Colorectal Cancer

### 4.1. Direct Visualization Test

**Figure 3:**
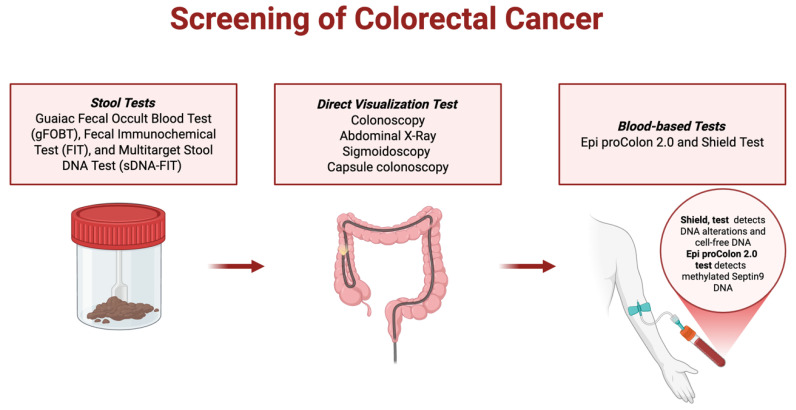
Methods for Screening Colorectal Cancer—A Comprehensive Visual Overview of Stool, Direct Visualization, and Blood-based Tests [45].

Direct visualization tests provide a thorough examination of the colon lining. Colonoscopy remains the most effective screening method, allowing physicians to identify and remove polyps while requiring sedation and thorough bowel cleansing. Virtual colonoscopy, which uses X-ray imaging, is less invasive but still requires bowel preparation, while sigmoidoscopy examines only the lower colon and rectum without sedation. Additional screening approaches include capsule colonoscopy, where a tiny camera inside a pill captures images of the digestive tract, and the double-contrast barium enema (DCBE), an older method using X-ray imaging with a barium solution [41] (Figure 3).

### 4.2. Stool Tests

CRC screening is essential for early detection and prevention. Various screening methods are available, each with its advantages and considerations. Stool tests, such as the Guaiac Fecal Occult Blood Test (gFOBT), Fecal Immunochemical Test (FIT), and Multitarget Stool DNA Test (sDNA-FIT), detect hidden blood or abnormal DNA markers in stool samples. A great advantage of this examination is its non-invasive nature and requires a lack of dietary restrictions, except for the gFOBT [41]. Stating that women are less likely to be diagnosed through screening and more likely to present as emergency cases. Screening tests like FIT may have lower sensitivity in women, potentially leading to more interval cancers, cancers diagnosed between screening tests. CRC screening tends to have lower sensitivity in women compared with men, but better specificity in women. As women are less likely than men to return a positive result, they are also less likely to undergo further investigation [42] (Figure 3).

### 4.3. Blood-Based Tests

Blood-based tests, such as Epi proColon 2.0 and Shield Test, detect cancer markers in the bloodstream. Although promising, these tests have not yet been fully incorporated into clinical guidelines [41]. The Food and Drug Administration (FDA) authorized the initial blood test for primary screening purposes in July 2024 for individuals with an average risk of colon cancer (Figure 3).

The Shield test is designed to detect the presence of specific changes to DNA that are circulating freely in the blood, known as cell-free DNA. These changes may indicate the presence of a tumor or precancerous growths in the colon. Based on the results of a study that involved nearly 8000 individuals, the test was approved for the detection of CRC in over 83% of the participants who were discovered to have CRC after undergoing a colonoscopy. However, its ability to identify precancerous growths in the colon was significantly diminished, with a sensitivity of only approximately 13% [43]. For the Epi proColon 2.0, the test comprises a qualitative assay for the polymerase chain reaction (PCR) detection of methylated Septin9 DNA, the presence of which is associated with CRC. However, positive results should be verified by colonoscopy or sigmoidoscopy [44] (Figure 3).

Choosing a screening test depends on factors such as individual risk level, invasiveness, preparation requirements, sedation needs, cost, and insurance coverage. Emerging technologies, including AI-assisted imaging and improved stool-based biomarkers, continue to advance CRC screening, enhancing its effectiveness and accessibility [41] (Figure 3).

## 5. Discussion

Sex-based differences in colorectal cancer represent a critical aspect of epidemiology, risk factors, tumor biology, and overall clinical outcomes. This comprehensive review highlights the significant disparities between male and female CRC patients, emphasizing the necessity of gender-specific prevention strategies, screening approaches, and therapeutic interventions.

One of the most crucial findings is the higher incidence and mortality rate of CRC among the male population. This discrepancy may be influenced by various factors, including hormonal differences, lifestyle factors, and genetic predispositions. As stated, males more frequently develop left-sided (distal) tumors, which respond better to conventional chemotherapy and targeted therapies such as anti-EGFR agents. Conversely, females tend to develop right-sided (proximal) tumors that exhibit aggressive characteristics, including MSI and BRAF mutations, often leading to poorer prognosis and reduced sensitivity to standard chemotherapy. These differences underline the need for sex-specific treatment approaches, particularly in cases of advanced-stage CRC.

Modifiable and non-modifiable risk factors play a critical role in shaping CRC development. While aging and hereditary predisposition contribute equally to CRC risk across sexes, modifiable factors such as obesity, smoking, and dietary habits exhibit distinct gender-dependent impacts. Obesity-related CRC risk is particularly pronounced in males with elevated body mass index (BMI), while central obesity (waist-to-hip ratio) serves as a stronger predictor in females. Similarly, smoking elevates CRC risk differently based on tumor location, with males more susceptible to distal colon cancer and females showing a stronger correlation with proximal colon and rectal cancer. These findings suggest the importance of gender-specific lifestyle recommendations in reducing the CRC burden.

Beyond lifestyle and genetic factors, immune response plays a pivotal role in CRC progression and treatment outcomes. Females generally exhibit stronger immune surveillance, characterized by higher CD8+ and CD4+ T cell infiltration. However, they also present elevated exhaustion markers, which could influence responses to immunotherapy. These sex-based immunological variations warrant further investigation to optimize immune-targeted therapies for CRC patients.

From a clinical perspective, tumor location significantly affects symptom presentation and diagnosis. Right-sided CRC, which is more common in females, often presents with vague symptoms such as anemia and abdominal pain, contributing to delayed diagnoses and worse survival outcomes. In contrast, left-sided CRC, prevalent in males, tends to cause more noticeable symptoms such as rectal bleeding and bowel obstruction, facilitating earlier detection. These differences highlight the necessity of tailoring screening strategies to enhance early CRC diagnosis in women.

Adding on a meta-analysis that was conducted aiming to examine the impact of gender on survival in CRC, analyzed data from 14 studies published between 1960 and 2017, revealing that females consistently had better overall (HR 0.87) and cancer-specific survival (HR 0.92) than males. Biological contributors to this disparity included higher rates of MSI in females, gender differences in EGFR signaling relevant to metastatic response, and distinct patterns in genetic markers such as TP53, BRAF, and EGFR. Hormonal influences, such as immunity linked to pregnancy, may also play a pivotal role. A sensitivity analysis that was performed confirmed robust findings with minimal publication bias, leading to the conclusion that gender is an independent prognostic factor, suggesting personalized approaches in colorectal cancer treatment and future research [46].

## 6. Conclusions

This review demonstrates that sex-based differences in colorectal cancer are multifactorial, encompassing genetic, immunologic, and clinical dimensions. Females benefit from the escape of select tumor-suppressor genes from X-inactivation, affording a level of redundancy that is absent in males, who consequently experience a higher overall cancer risk. Additionally, differences in the tumor microenvironment are evident, with females exhibiting increased CD4+ and CD8+ T cell infiltration and distinct gene expression profiles that may influence tumor aggressiveness and therapeutic response. Clinically, the predominance of right-sided tumors in females versus left-sided tumors in males further accentuates these disparities, as tumor location bears on detection, prognosis, and treatment efficacy. Furthermore, variances in risk factors such as body mass index and tobacco smoking contribute to the complex epidemiologic and pathologic profiles observed between sexes.

Future research should focus on elucidating the underlying molecular and immunologic mechanisms that drive these sex-specific differences in CRC to facilitate the development of personalized screening and treatment strategies. Integrating genomic, proteomic, and immunologic data will be critical in identifying reliable sex-specific biomarkers and refining risk stratification models. There is a clear need for prospective studies to assess how tailored interventions based on these differences could improve early detection and therapeutic outcomes. Ultimately, bridging bench research with clinical application promises to enhance precision medicine approaches and optimize management protocols for both male and female CRC patients.

## Figures and Tables

**Table 2:** Sex-Based Immune Differences in Colorectal Cancer Tumor Microenvironment—A comparison of immune cell infiltration and gene expression between male and female CRC patients, highlighting sex-specific influences on tumor progression and immune response.

Immune Factor	Females (Higher Expression)	Males (Lower Expression)	Implications
CD4+ T Cells	More prevalent in tumor and metastatic lymph nodes	Less infiltration	Stronger immune surveillance in females
CD8+ T Cells	Higher in uninvolved colon and early-stage CRC	Lower, especially in advanced-stage CRC	Correlated with improved survival
T Cell Activation Genes	IL18R1, STAT4, GBP1, GZMK	Lower expression	Enhanced immune response in females
Regulatory T Cells (Tregs)	Lower expression (DAB2, TNFRSF25, LRRC32)	Higher expression	Reduced immune suppression in females
Exhaustion Markers	PD-L1, TIGIT, FBP1 (higher in females)	Lower expression	Possible influence on response to immunotherapy

**Table 3:** Tumor Location Differences and Their Implications Based on Sex. This table highlights the prevalence, molecular features, treatment response, and prognosis variations between right-sided and left-sided colorectal cancer in male and female patients, emphasizing sex-specific clinical outcomes.

Tumor Location	Prevalence	Molecular Features	Treatment Response	Prognosis
Right-Sided (Proximal) Colorectal Cancer (CRC)	More common in females	High MSI, BRAF mutations	Less responsive to chemotherapy, better suited for immunotherapy	Generally worse due to late diagnosis
Left-Sided (Distal) CRC	More common in males	CIN, APC, TP53, KRAS mutations	Responds well to chemotherapy and targeted therapies (EGFR inhibitors)	Better prognosis, earlier diagnosis possible

**Table 4:** Symptomatology of Colorectal Cancer by Tumor Location and Pathophysiology. This table presents the distinct symptom profiles of left-sided and right-sided colorectal cancer (CRC) based on underlying pathophysiological mechanisms.

Tumor Location	Symptom Type	Common Symptoms	Clinical Implications
Left-Sided (Distal) Colorectal Cancer (CRC)	Gastrointestinal	Constipation, change in bowel habits, narrow/ribbon-shaped stool	Tumor often encircles the bowel wall, leading to partial obstruction
	Hematological	Rectal bleeding	More noticeable blood loss due to proximity to the rectum
Right-Sided (Proximal) CRC	Gastrointestinal	Abdominal pain, vague symptom presentation	Tumors grow into the colonic space rather than obstructing the lumen
	Hematological	Anemia, occult blood in stool	Chronic blood loss may lead to iron-deficiency anemia
Advanced Stage Symptoms	Systemic	Unexplained weight loss, fatigue	Indicates tumor progression and potential metastatic disease

**Table 5:** Sex-Based Differences in Colorectal Cancer Prevalence and Risk Factors. This table highlights key distinctions in tumor characteristics, age of onset, mortality trends, BMI influence, and smoking-related risks between male and female patients.

Factor	Females	Males	Implications
Colorectal Cancer (CRC) Prevalence	Higher rates of right-sided CRC (59.2%)	Higher rates of left-sided CRC (49.3%)	Tumor location affects diagnosis and treatment response
Age at Diagnosis	Median age: 72 (colon), 69 (rectal)	Median age: 71 (colon), 68 (rectal)	Males tend to be diagnosed younger
Mortality Trends	CRC is the 2nd leading cause of cancer death < 50 yrs	CRC is the leading cause of cancer death < 50 yrs	Younger populations require urgent screening strategies
Tumor Characteristics	More poorly differentiated tumors (13.9%)	Lower rates of poor differentiation (9.4%)	Molecular differences impact therapy effectiveness
Microsatellite Instability (MSI)	Higher MSI rates (22.9%)	Lower MSI rates (12.0%)	MSI tumors may respond better to immunotherapy in females
Body Mass Index (BMI)	Higher waist-to-hip ratio (WHR) increases CRC risk (25%)	Higher BMI increases CRC risk (23%)	Central obesity is more predictive for females
Smoking and CRC Risk	Higher Risk for right-sided colon and rectal cancer	Higher Risk for left-sided colon cancer	Sex-specific smoking risks influence prevention strategies
